# Emergence of Chiral Defective Pores Through Chiral Linker Exchange in Nonchiral MOFs for Enantioselective Recognition

**DOI:** 10.1002/anie.8824438

**Published:** 2026-06-30

**Authors:** Zongsu Han, Kun‐Yu Wang, Jiatong Huo, Joshua Rushlow, Zhaoyi Liu, Yihao Yang, Rong‐Ran Liang, Wei Shi, Hong‐Cai Zhou

**Affiliations:** ^1^ Department of Chemistry Texas A&M University Texas USA; ^2^ Frontiers Science Center for New Organic Matter State Key Laboratory of Elemento‐Organic Chemistry and Department of Chemistry College of Chemistry Nankai University Tianjin China

**Keywords:** chiral defect engineering, enantioselective recognition, linker exchange, metal–organic framework

## Abstract

Enantioselective recognition is vital for numerous chemical and biological applications, which, however, remains challenging due to the nearly indistinguishable physicochemical properties of enantiomers. In this study, we report a luminescent sensing strategy for enantioselective recognition based on metal‐organic frameworks (MOFs) constructed through chiral linker exchange, which simultaneously introduces chirality and defective sites into the frameworks. The resulting chiral defective MOFs exhibit confined nanopore environments, resulting in distinct luminescence responses toward enantiomers. A pair of enantiomeric MOFs was constructed, exhibiting opposite selective recognition performance toward R‐ and S‐substrates. The sensing behavior arises from the interplay of competitive absorption and electron transfer process, while disparities in binding affinities serve as the dominating factor dictating the enantioselectivity. Meanwhile, this system enables the quantitative detection of enantiomeric excess (ee) values in mixtures through differential luminescence responses. Due to its facile synthesis routes, selectivity, and ease of implementation, this strategy offers a practical approach for developing chiral luminescent sensing materials, while highlighting the significance of host‐guest interactions in sensing.

## Graphical abstract

Chiral recognition remains challenging due to minimal property differences. This work develops chiral defective MOFs via linker exchange to enable rapid, direct, luminescent enantioselective sensing, offering a simple, modular strategy that broadens MOF functionality for portable, low‐cost applications in pharmaceuticals, food safety, and environmental monitoring.

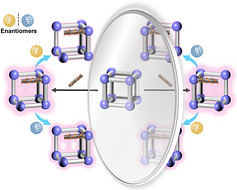

## 1 Introduction

Chirality is ubiquitous in nature and plays a critical role in chemical and biological systems [1, 2, 3]. Chiral molecules are widely used in modern industries, ranging from pharmaceuticals to agrochemicals [4, 5]. Instantaneous chiral recognition is particularly critical in medicinal and industrial contexts, where the activity, efficacy, or safety of chiral compounds can differ dramatically between enantiomers [6, 7, 8]. However, despite its prevalence, the recognition of enantiomers remains a significant challenge due to their nearly identical physicochemical properties. Traditional enantioselective analysis methods typically require sophisticated instrumentation, lengthy procedures, and specialized personnel, which restricts their applicability in the most crucial scenarios, such as daily usage and real time industrial monitoring.

Luminescent sensing, benefiting from its real time, efficient, and sensitive detection capabilities, has been widely validated across various fields, including environmental monitoring, biochemical analysis, and industrial recognition [9, 10, 11, 12]. Luminescence signals provide direct visual readouts and enable high‐throughput or real time measurements without the need for complex instrumentation and procedure. Compared with conventional analytical methods, luminescent sensing is more amenable to miniaturization and on‐site deployment, making it particularly suitable for real time monitoring in industrial processing, pharmaceutical pipelines, and environmental remediation [13, 14, 15, 16, 17, 18].

Metal–organic frameworks (MOFs), due to their structural diversity, tunable porosity, and chemical modularity [19, 20, 21, 22, 23], have become an emerging class of materials for luminescent sensing [24, 25, 26, 27, 28, 29]. MOFs have been successfully applied in gas sensing, water quality testing, and biomarker detection, attributed to their various functional luminescent centers and designable pore environments. However, the development of MOF‐based sensing materials for chiral molecules is still in its early stages. Conventional strategies, such as directly incorporating chiral ligands or inducing chirality during MOF synthesis, often encounter synthetic difficulty and limited predictability, necessitating extensive trial‐and‐error attempts [30, 31]. Targeted modification approaches, such as guest exchange and coordination functionalization, have been explored previously, but these methods usually reduce the pore size, thereby restricting the accessible substrate range [32, 33, 34]. Larger ligands can partially mitigate this issue [32], but they will inevitably increase both the synthetic complexity and the overall cost.

**SCHEME 1:**
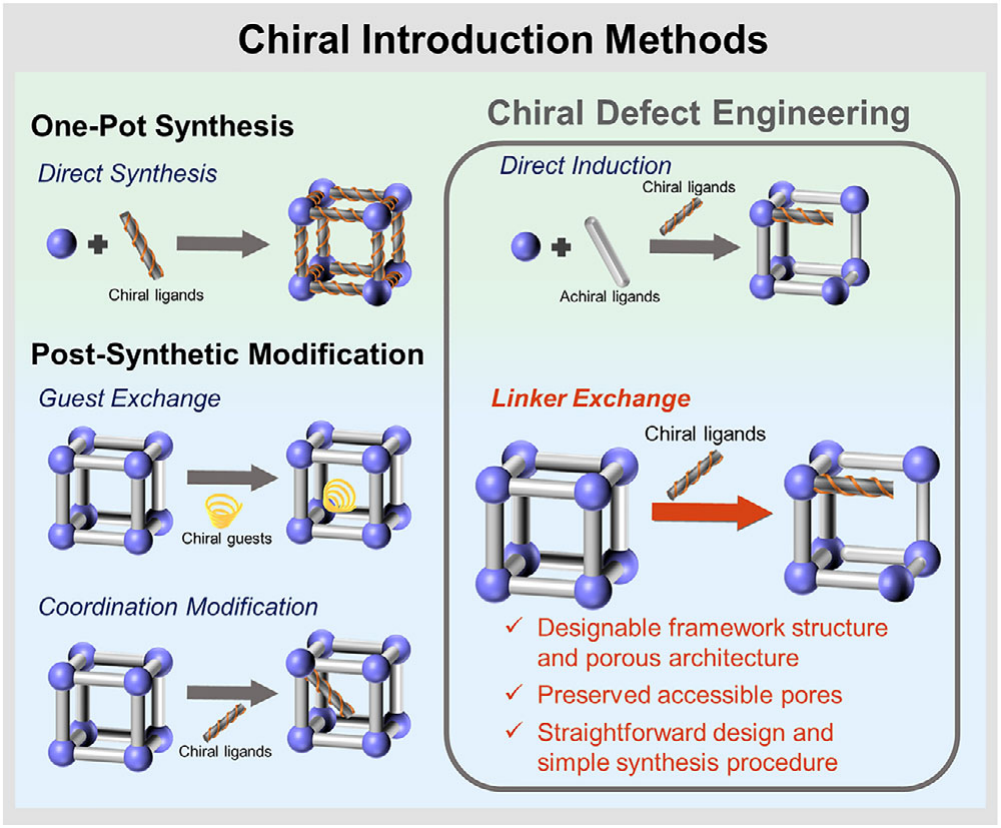
Comparisons of chiral defect engineering through linker exchange with other methods to construct MOF‐based enantioselective sensing materials.

Chiral defect engineering offers a promising solution by simultaneously introducing chiral centers and maintaining or even enlarging the accessible pores, thus circumventing the common trade‐off between functionality and porosity. Nevertheless, one‐pot syntheses demand exceptionally high framework stability and tunable crystallization kinetics, as MOFs must withstand the presence of chiral ligands and nucleate at an appropriate rate to successfully form the crystalline structure [35]. To overcome these obstacle, in this work, we develop a chiral linker exchange strategy to endow a nonchiral Eu‐FDA framework [36], which is sensitive to synthetic conditions, with both chirality and defective pores (Scheme 1). Through partial substitution of 2,5‐furandicarboxylic (FDA^2−^) with R‐/S‐2‐tetrahydrofurancarboxylic (R‐/S‐TFA^−^), the resulting enantiomeric pair Eu‐FDA‐R/S exhibits mirrored enantioselective luminescence responses toward the corresponding R‐ and S‐substrates, as well as enabling quantitative recognition of the enantiomeric excess (ee) values in the mixed samples. Due to its designable framework structures, preserved accessible pores, and straightforward synthesis route, this strategy offers a practical and facile route for constructing chiral luminescent sensing materials for various chiral analytes.

## 2 Results and Discussion

**FIGURE 1:**
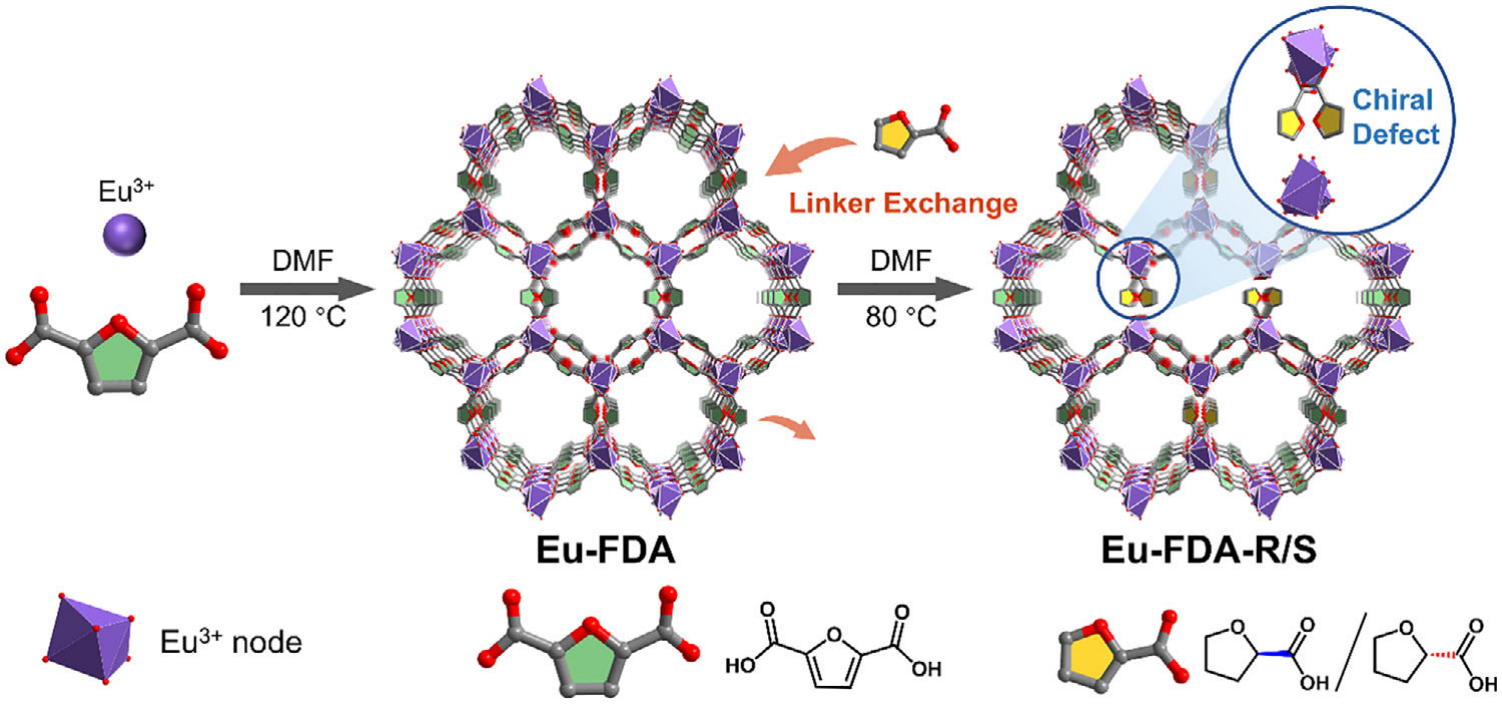
Construction pathway for Eu‐FDA‐R/S by chiral linker exchange.

Pristine Eu‐FDA was obtained by the reaction of Eu(NO_3_)_3_·6H_2_O and H_2_FDA in *N*,*N*‐dimethylformamide (DMF) under 120°C [36]. Eu^3+^ is selected due to its characteristic sharp emissions, while FDA^2−^ for its highly efficient “antenna effect”. Then, DMF solutions of R‐/S‐HTFA were added to Eu‐FDA under 80°C, respectively, to give Eu‐FDA‐R/S (Figure 1). No discernible linker exchange process occurs at room temperature, indicating that the process is synergistically driven by elevated temperature and ligand concentration. R‐/S‐HTFA were chosen due to their appropriate size and geometry relative to H_2_FDA, good solubility in DMF, and structural compatibility with the framework without inducing obvious structural collapse. Phase purities of these MOFs were confirmed by powder X‐ray diffraction (PXRD) patterns (Figure S1). To confirm the successful introduction and the exchange ratio of the chiral centers, the samples were first washed and soaked in DMF overnight, to remove any free HTFA in the pores. Then the samples were washed and soaked in CH_2_Cl_2_ overnight, to remove the free DMF in the pores. ^1^H nuclear magnetic resonance (NMR) spectra of the digested samples confirmed the successful introduction of R‐/S‐HTFA, while the compositions changed from 4.5 FDA^2−^ and 3 DMF (Eu_2_(FDA)_3_(DMF)_2_ from single crystal X‐ray diffraction analysis) to 3.5 FDA^2−^, 1 TFA^−^ and 3 DMF after linker exchange process (Figures S2 and S3), confirming that ∼22% FDA^2−^ but not DMF were exchanged by TFA^−^. Thermogravimetric analysis showed that these frameworks undergo almost no change in weight until 200°C (Figure S4). As shown in the time‐resolved luminescence profiles in DMF (Figures S5 and S6), the emission intensities of these MOFs exhibit only minor variations, demonstrating their robustness under sensing conditions. Variations in the pore environment were characterized by liquid‐phase iodine adsorption characterized by ultraviolet–visible (UV–vis) spectroscopy (Figures S7–S9). Enhancements in both adsorption kinetics and capacity (Figure S10) indicate that the TFA^−^ incorporation expands the available pore volume, confirming the generation of the defective pores.

To study the enantioselective sensing functions of Eu‐FDA‐R/S, chiral molecules R‐/S‐2‐amino‐1‐butanol, R‐/S‐2‐methylpiperazine, R‐/S‐carvone, and quinine and quinidine (Figure S11), with various sizes and shapes, were selected as the target species. Response time of Eu‐FDA‐R/S toward these enantiomers was within 1 min (Figures S12–S27), which can be used for instant sensing applications. For the sensing toward the enantiomers, the emission intensities of Eu‐FDA‐R/S distinctly decreased with the gradual addition of the analytes (Figures S28–S43), and the quenching efficiencies can be quantitatively described by the linear Stern–Volmer (S–V) equation of *I*_0_/*I* = *K*_SV_[*C*]+b [37] (Figures S44–S51), where *K*_SV_ is the quenching constant (M^−1^), [*C*] is the concentration of the analyte, b is a constant, and *I*_0_ and *I* are the emission intensities before and after the addition of the analyte, respectively. Quenching efficiencies and the ratios toward enantiomers by Eu‐FDA‐R/S are summarized and shown in Figure 2. For Eu‐FDA‐R, the *K*_SV_ values for R‐2‐amino‐1‐butanol, S‐2‐methylpiperazine, S‐carvone, and quinidine are clearly higher than those of their enantiomers. For Eu‐FDA‐S, the *K*_SV_ values are higher for the opposite enantiomers, demonstrating a reversed enantioselectivity compared to Eu‐FDA‐R. For comparison, Eu‐FDA is also studied using these analytes. The emission intensities of Eu‐FDA also distinctly decreased with the gradual addition of these analytes (Figures S52–S59), but showed no obvious enantioselectivity toward the enantiomers (Figures S60–S63). These results indicate that the enantioselective sensing property arises from the chiral defects, which create guest‐accessible sites capable of accommodating chiral molecules, an ability absent in the pristine MOF.

**FIGURE 2:**
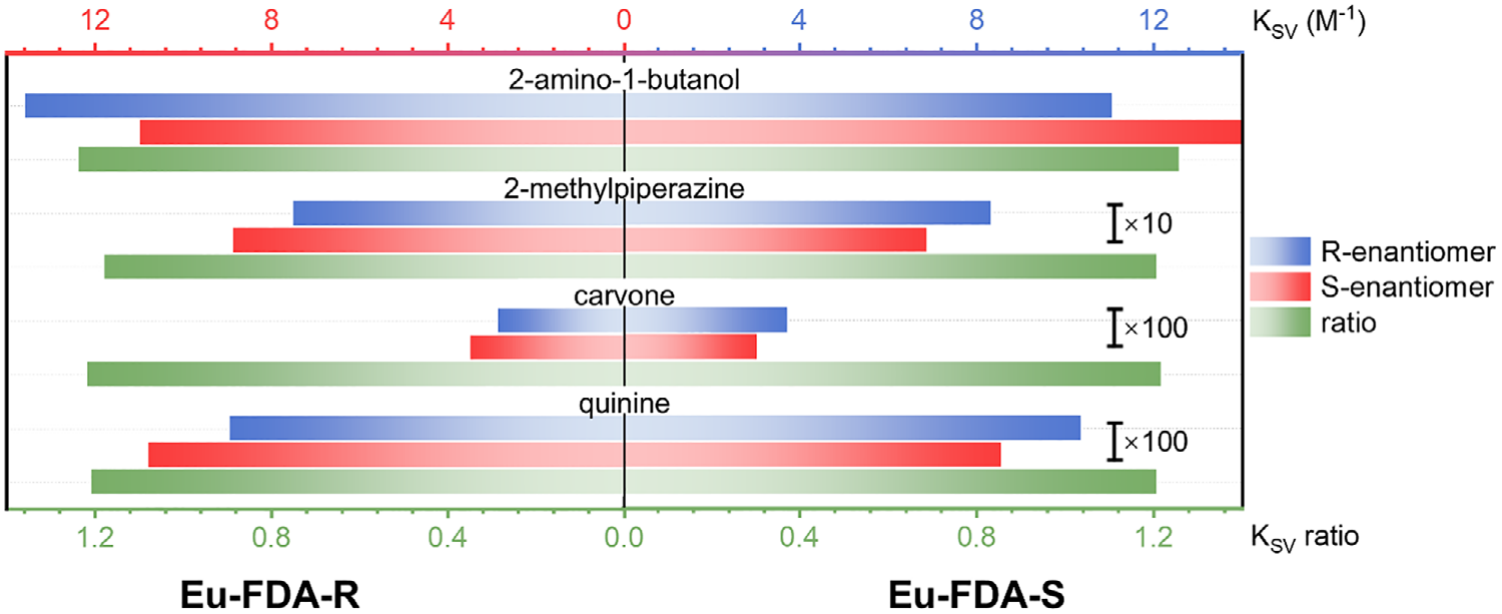
Quenching efficiencies and the ratios of Eu‐FDA‐R/S toward various analytes. Blue columns and red columns represent the *K*_SV_ values of Eu‐FDA‐R/S toward R‐/S‐enantiomer, respectively. Green columns represent the ratios of the higher to lower *K*_SV_ values for each pair of target enantiomers. X10 and X100 in the figure indicate the order of magnitude of the *K*_SV_ values.

For the enantiomer mixtures, the ee values can be further obtained based on the different quenching behaviors of Eu‐FDA‐R/S toward the analytes (Figure 3). The quantity ee is defined as (*a*–*b*)/(*a*+*b*)×100%, where *a* and *b* are the concentrations of different enantiomers. The total concentration (*a*+*b*) can be calculated by *m*/*MV*, where *m* is the mass of the analyte, *M* is the molecular mass of the analyte, and *V* is the volume of the solution. The emission spectra of Eu‐FDA‐R/S upon the addition of mixtures with different proportions were measured (Figures S64–S87). The *I*_0_/*I* ratios of Eu‐FDA‐R/S changed linearly as the ee values increased (Figure 3), while Eu‐FDA‐R and Eu‐FDA‐S exhibited almost mirror‐image responses.

**FIGURE 3:**
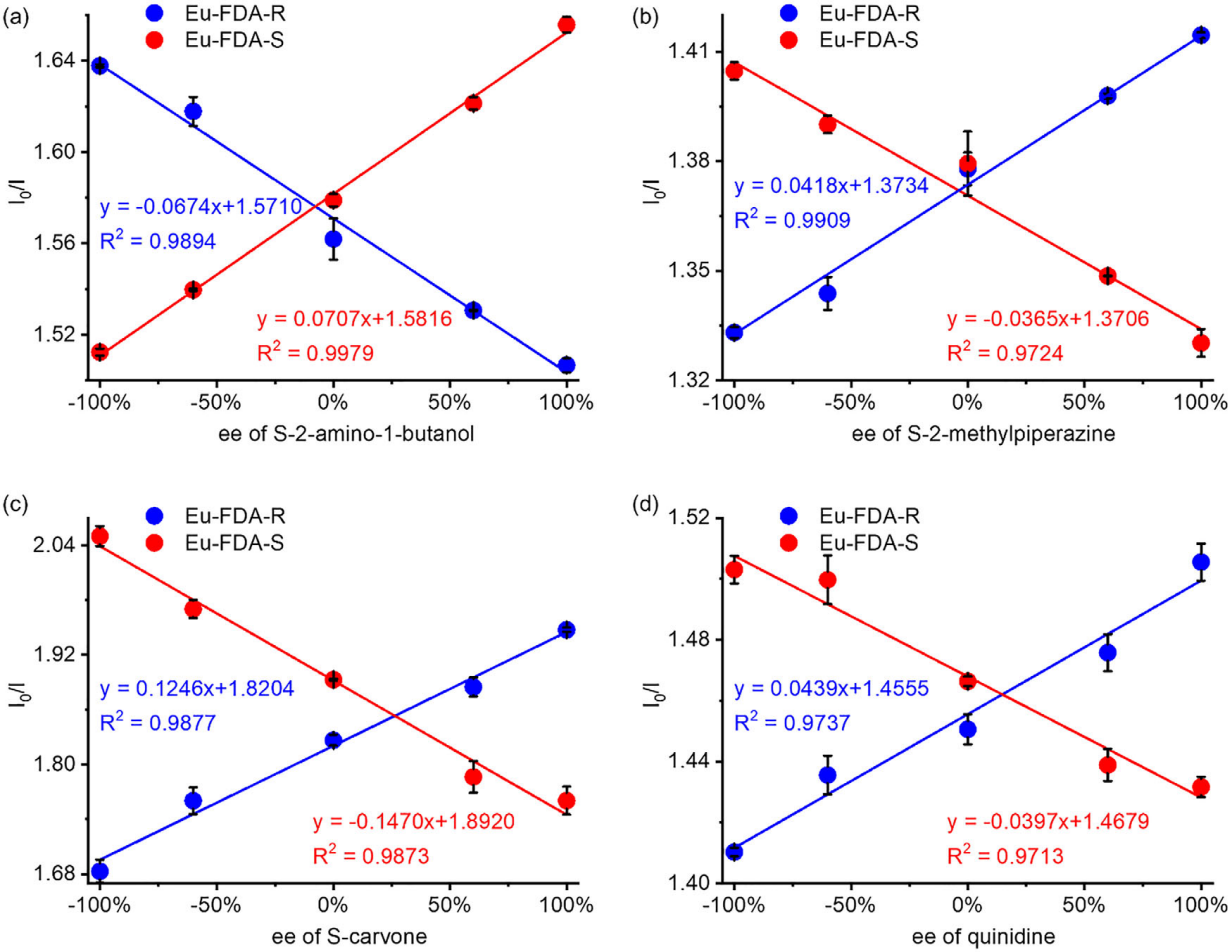
Emission intensity changes of Eu‐FDA‐R/S with different ee values of (a) R‐/S‐2‐amino‐1‐butanol, (b) R‐/S‐2‐methylpiperazine, (c) R‐/S‐carvone, and (d) quinine and quinidine.

A series of characterizations was conducted to further understand the mechanism behind the quenching response of Eu‐FDA‐R/S toward these enantiomers. PXRD patterns and infrared (IR) spectra of Eu‐FDA‐R/S before and after sensing did not change obviously, revealing no structural transformation or collapse during the sensing process (Figures S88 and S89). For the emission energy, there are no overlaps between the emission spectra of Eu‐FDA‐R/S and the UV–vis spectra of the analytes (Figure S90), suggesting that the Förster resonance energy transfer (FRET) mechanism is absent [38].

**FIGURE 4:**
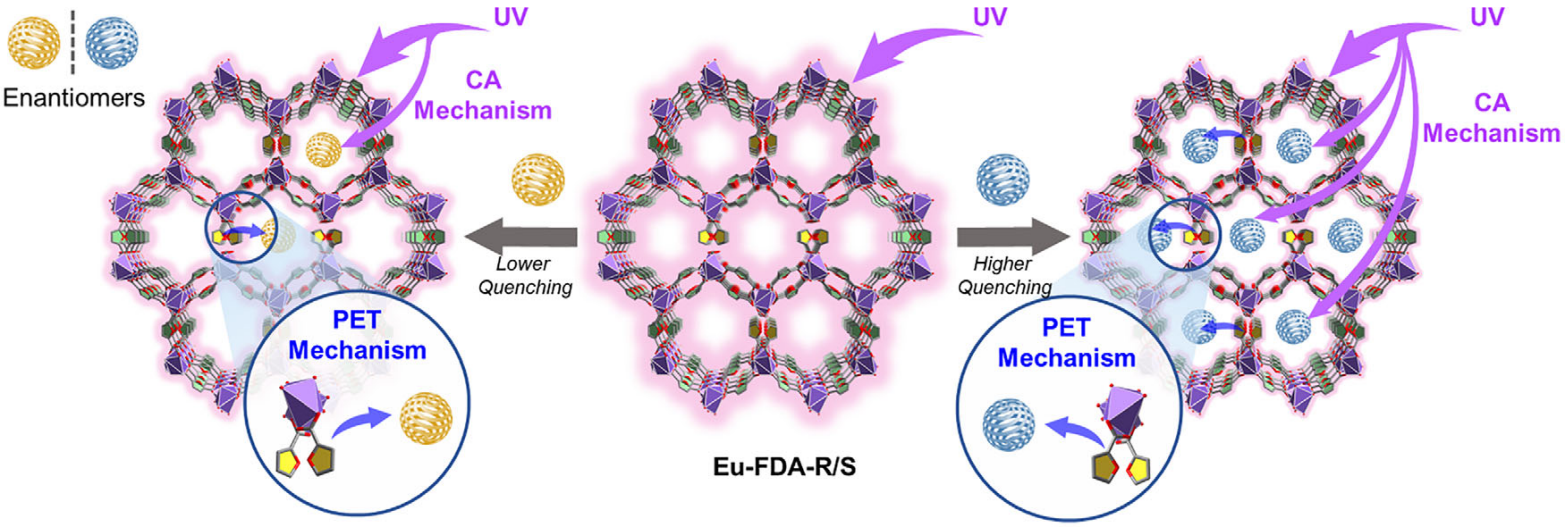
Sensing mechanism and selective sensing mechanism for Eu‐FDA‐R/S toward enantiomers. PET stands for photoinduced electron transfer, whereas CA stands for competitive absorption.

For the excitation energy, the UV–vis absorption spectra of Eu‐FDA‐R/S overlapped well with the enantiomers in the range of 240–280 nm (Figure S91), indicating that competitive absorption (CA) of the excitation energy occurs between the enantiomers and Eu‐FDA‐R/S, inducing a quenching response [38]. Furthermore, for the excited state, the lowest unoccupied molecular orbital (LUMO) energy levels of HTFA were lower than 2‐amino‐1‐butanol and 2‐methylpiperazine, but higher than carvone and quinine (Figure S92), indicating the absence of photoinduced electron transfer (PET) mechanism toward the former two enantiomers, while it exists for carvone and quinine [38]. This extra mechanism may partly contribute to the higher quenching efficiencies toward carvone and quinine compared with 2‐amino‐1‐butanol and 2‐methylpiperazine. In summary, the quenching response of Eu‐FDA‐R/S toward 2‐amino‐1‐butanol and 2‐methylpiperazine originates from the CA of the excited energy, while the quenching response toward carvone and quinine is induced by both CA and PET processes (Figure 4).

Regarding enantioselectivity, adsorption experiments of Eu‐FDA‐R/S toward the enantiomers were conducted under the same conditions and evaluated by ^1^H NMR spectra (Figures S93–S96). While the adsorption capacities of Eu‐FDA‐R toward R‐2‐amino‐1‐butanol, S‐2‐methylpiperazine, S‐carvone, and quinidine are higher than those of their enantiomers within the same time period, which gives the opposite tendency by Eu‐FDA‐S, showing the reversed enantioselectivity relative to Eu‐FDA‐R. These results indicate that the enantioselective sensing performance comes from their selective adsorption capacities.

## 3 Conclusion

In summary, a versatile luminescent sensing strategy based on MOFs constructed through chiral linker exchange was reported, which effectively integrates chirality and structural defects within a single platform. The resulting defective MOFs exhibit chiral pore environments that enable distinct luminescence responses toward enantiomeric molecules. A pair of enantiomeric MOFs constructed using R‐/S‐HTFA were found to display opposite enantioselective recognition behaviors toward R‐ and S‐substrates and allowed for reliable detection of ee values in their mixed samples. Furthermore, the sensing performance is induced by both competitive absorption and electron transfer processes, while the observed enantioselectivity primarily arises from variations in adsorption affinity. These results indicate that chiral linker exchange is a general and efficient approach for constructing multifunctional MOFs and provide a facile and sensitive method for enantioselective recognition. Moreover, this work expands the application of post‐synthetic MOF modification by demonstrating the potential of linker exchange for the rational design of advanced chiral MOF‐based sensing materials. The combination of chirality and defect engineering opens opportunities for developing multi‐responsive and highly selective functional systems, which may find applications in enantiomeric analysis, catalysis, and beyond.

## Author Contributions

**Zongsu Han**: writing – original draft, investigation, conceptualization, data curation, formal analysis. **Kun‐Yu Wang**: writing – review and editing, visualization, investigation. **Jiatong Huo**: visualization. **Joshua Rushlow**: writing – review and editing. **Zhaoyi Liu**: visualization. **Yihao Yang**: writing – review and editing, software. **Rong‐Ran Liang**: writing – review and editing. **Wei Shi**: writing – review and editing, supervision, funding acquisition. **Hong‐Cai Zhou**: writing – review and editing, supervision, funding acquisition.

## Conflicts of Interest

The authors declare no conflicts of interest.

## Supporting information




**Supporting File**: anie72646‐sup‐0001‐SuppMat.docx.

## Data Availability

The data that supports the findings of this study are available in the Supporting Information of this article.
