# Supplementary material for: Emergence of Chiral Defective Pores Through Chiral Linker Exchange in Nonchiral MOFs for Enantioselective Recognition
Source: Angew Chem Int Ed Engl. 2026 Jun 30;65(33):e8824438. doi: 10.1002/anie.8824438 (PMC13452546; doi:10.1002/anie.8824438)
Supplement: Supplementary file 1 [file ANIE-65-e8824438-s001.docx]

Supporting Information

**Emergence of Chiral Defective Pores through Chiral Linker Exchange in Nonchiral MOFs for Enantioselective Recognition**

Zongsu Han,^[a]^ Kun-Yu Wang,^[a]^ Jiatong Huo,^[a]^ Joshua Rushlow,^[a]^ Zhaoyi Liu,^[a]^ Yihao Yang,^[a]^ Rong-Ran Liang,^[a]^ Wei Shi,^[b],^* and Hong-Cai Zhou^[a],^*

^[a]^Department of Chemistry, Texas A&M University, College Station, Texas 77843, United States. E-mail: zhou@chem.tamu.edu.

^[b]^Frontiers Science Center for New Organic Matter, State Key Laboratory of Elemento-Organic Chemistry and Department of Chemistry, College of Chemistry, Nankai University, Tianjin 300071, China. E-mail: shiwei@nankai.edu.cn.

| Contents | |
| --- | --- |
| Methods | 2 |
| Structures and Basic Characterizations | 3 |
| Luminescence Sensing | 8 |
| Sensing Mechanism | 40 |
| References | 45 |

**Methods**

**Materials and methods**

All reagents were commercially available and used without further purification. ^1^H NMR spectra were recorded on a Bruker Avance NEO 400 NMR spectrometer. PXRD measurements were performed using a Bruker Powder-ECO X-ray diffractometer with Cu-Kα radiation. Luminescence spectra were recorded on a Horiba Fluoromax-4 fluorescence spectrophotometer. IR spectra were tested by a Shimadzu IRAffinity-1S FTIR spectrometer. UV-vis absorption spectra were measured by a Shimadzu UV-2450 absorption spectrometer. For ^1^H NMR tests, 3 mg linkers were dissolved in 0.5 mL *d*_6_-dimethyl sulfoxide (DMSO), while 3 mg MOFs were dissolved in 0.5 mL *d*_6_-DMSO and 5 μL D_2_SO_4_. Further details regarding the methods can be found in the supplemental information.

**Synthesis**

*Eu-FDA* was synthesized according to the literature.^1,2^ 44.6 mg (0.1 mmol) Eu(NO_3_)_3_·6 H_2_O, 31.2 mg (0.2 mmol) H_2_FDA, and 2 mL DMF were added in a 20 mL sealed glass bottle under ultrasound, and then heated at 120 °C for one day. Then the crystals were collected by filtration, washed with fresh DMF, and soaked in fresh DMF for night.

*Eu-FDA-R/S* were synthesized through the linker exchange towards Eu-FDA. 20 mg Eu-FDA were first ground into fine powder to facilitate the linker exchange process, and soaked in the mixture of 0.2 mL R-/S-HTFA and 10 mL DMF at 80 °C for one week. Then the samples were collected by filtration, washed with fresh DMF, and soaked in fresh DMF for night.

**Luminescence experiments**

All samples were grinded into fine powder before use. The samples for luminescence experiments were dispersed in water by ultrasound for 10 minutes to form a clear suspension with a concentration of 0.3 mg mL^-1^. Each line was tested after 10 seconds ultrasound.

**Structures and Basic Characterizations**

**Figure S1.** PXRD patterns of simulated and as-synthesized Eu-FDA-R, Eu-FDA-S and Eu-FDA.

**Figure S2.** Liquid ^1^H NMR spectra of Eu-FDA, Eu-FDA-R and R-HTFA.

**Figure S3.** Liquid ^1^H NMR spectra of Eu-FDA, Eu-FDA-S and S-HTFA.

**Figure S4.** TGA curves of Eu-FDA, Eu-FDA-R and Eu-FDA-S.

**Figure S5.** Emission spectra and intensity changes of Eu-FDA-R with time.

**Figure S6.** Emission spectra and intensity changes of Eu-FDA-S with time.

**Figure S7.** UV-vis absorption spectra of I_2_ methanol solution with 5.0 mg Eu-FDA.

**Figure S8.** UV-vis absorption spectra of I_2_ methanol solution with 5.0 mg Eu-FDA-R.

**Figure S9.** UV-vis absorption spectra of I_2_ methanol solution with 5.0 mg Eu-FDA-S.

**Figure S10.** UV-vis absorption intensity changes at 443 nm of I_2_ methanol solution with 5.0 mg MOFs.

**Luminescence Sensing**

**Figure S11.** Structures of the chiral analytes.

**Figure S12.** Emission spectra and intensity changes of Eu-FDA-R with R-2-amino-1-butanol.

**Figure S13.** Emission spectra and intensity changes of Eu-FDA-R with S-2-amino-1-butanol.

**Figure S14.** Emission spectra and intensity changes of Eu-FDA-S with R-2-amino-1-butanol.

**Figure S15.** Emission spectra and intensity changes of Eu-FDA-S with S-2-amino-1-butanol.

**Figure S16.** Emission spectra and intensity changes of Eu-FDA-R with R-2-methylpiperazine.

**Figure S17.** Emission spectra and intensity changes of Eu-FDA-R with S-2-methylpiperazine.

**Figure S18.** Emission spectra and intensity changes of Eu-FDA-S with R-2-methylpiperazine.

**Figure S19.** Emission spectra and intensity changes of Eu-FDA-S with S-2-methylpiperazine.

**Figure S20.** Emission spectra and intensity changes of Eu-FDA-R with R-carvone.

**Figure S21.** Emission spectra and intensity changes of Eu-FDA-R with S-carvone.

**Figure S22.** Emission spectra and intensity changes of Eu-FDA-S with R-carvone.

**Figure S23.** Emission spectra and intensity changes of Eu-FDA-S with S-carvone.

**Figure S24.** Emission spectra and intensity changes of Eu-FDA-R with quinine.

**Figure S25.** Emission spectra and intensity changes of Eu-FDA-R with quinidine.

**Figure S26.** Emission spectra and intensity changes of Eu-FDA-S with quinine.

**Figure S27.** Emission spectra and intensity changes of Eu-FDA-S with quinidine.

**Figure S28.** Emission spectra of Eu-FDA-R with the additions of R-2-amino-1-butanol.

**Figure S29.** Emission spectra of Eu-FDA-R with the additions of S-2-amino-1-butanol.

**Figure S30.** Emission spectra of Eu-FDA-S with the additions of R-2-amino-1-butanol.

**Figure S31.** Emission spectra of Eu-FDA-S with the additions of S-2-amino-1-butanol.

**Figure S32.** Emission spectra of Eu-FDA-R with the additions of R-2-methylpiperazine.

**Figure S33.** Emission spectra of Eu-FDA-R with the additions of S-2-methylpiperazine.

**Figure S34.** Emission spectra of Eu-FDA-S with the additions of R-2-methylpiperazine.

**Figure S35.** Emission spectra of Eu-FDA-S with the additions of S-2-methylpiperazine.

**Figure S36.** Emission spectra of Eu-FDA-R with the additions of R-carvone.

**Figure S37.** Emission spectra of Eu-FDA-R with the additions of S-carvone.

**Figure S38.** Emission spectra of Eu-FDA-S with the additions of R-carvone.

**Figure S39.** Emission spectra of Eu-FDA-S with the additions of S-carvone.

**Figure S40.** Emission spectra of Eu-FDA-R with the additions of quinine.

**Figure S41.** Emission spectra of Eu-FDA-R with the additions of quinidine.

**Figure S42.** Emission spectra of Eu-FDA-S with the additions of quinine.

**Figure S43.** Emission spectra of Eu-FDA-S with the additions of quinidine.

**Figure S44.** Intensity changes of Eu-FDA-R with the additions of 2-amino-1-butanol.

**Figure S45.** Intensity changes of Eu-FDA-S with the additions of 2-amino-1-butanol.

**Figure S46.** Intensity changes of Eu-FDA-R with the additions of 2-methylpiperazine.

**Figure S47.** Intensity changes of Eu-FDA-S with the additions of 2-methylpiperazine.

**Figure S48.** Intensity changes of Eu-FDA-R with the additions of carvone.

**Figure S49.** Intensity changes of Eu-FDA-S with the additions of carvone.

**Figure S50.** Intensity changes of Eu-FDA-R with the additions of quinine and quinidine.

**Figure S51.** Intensity changes of Eu-FDA-S with the additions of quinine and quinidine.

**Figure S52.** Emission spectra of Eu-FDA with the additions of R-2-amino-1-butanol.

**Figure S53.** Emission spectra of Eu-FDA with the additions of S-2-amino-1-butanol.

**Figure S54.** Emission spectra of Eu-FDA with the additions of R-2-methylpiperazine.

**Figure S55.** Emission spectra of Eu-FDA with the additions of S-2-methylpiperazine.

**Figure S56.** Emission spectra of Eu-FDA with the additions of R-carvone.

**Figure S57.** Emission spectra of Eu-FDA with the additions of S-carvone.

**Figure S58.** Emission spectra of Eu-FDA with the additions of quinine.

**Figure S59.** Emission spectra of Eu-FDA with the additions of quinidine.

**Figure S60.** Intensity changes of Eu-FDA with the additions of 2-amino-1-butanol.

**Figure S61.** Intensity changes of Eu-FDA with the additions of 2-methylpiperazine.

**Figure S62.** Intensity changes of Eu-FDA with the additions of carvone.

**Figure S63.** Intensity changes of Eu-FDA with the additions of quinine and quinidine.

**Figure S64.** Emission spectra of Eu-FDA-R with -60% ee S-2-amino-1-butanol.

**Figure S65.** Emission spectra of Eu-FDA-R with 0% ee S-2-amino-1-butanol.

**Figure S66.** Emission spectra of Eu-FDA-R with 60% ee S-2-amino-1-butanol.

**Figure S67.** Emission spectra of Eu-FDA-S with -60% ee S-2-amino-1-butanol.

**Figure S68.** Emission spectra of Eu-FDA-S with 0% ee S-2-amino-1-butanol.

**Figure S69.** Emission spectra of Eu-FDA-S with 60% ee S-2-amino-1-butanol.

**Figure S70.** Emission spectra of Eu-FDA-R with -60% ee S-2-methyipiperazine.

**Figure S71.** Emission spectra of Eu-FDA-R with 0% ee S-2-methyipiperazine.

**Figure S72.** Emission spectra of Eu-FDA-R with 60% ee S-2-methyipiperazine.

**Figure S73.** Emission spectra of Eu-FDA-S with -60% ee S-2-methyipiperazine.

**Figure S74.** Emission spectra of Eu-FDA-S with 0% ee S-2-methyipiperazine.

**Figure S75.** Emission spectra of Eu-FDA-S with 60% ee S-2-methyipiperazine.

**Figure S76.** Emission spectra of Eu-FDA-R with -60% ee S-carvone.

**Figure S77.** Emission spectra of Eu-FDA-R with 0% ee S-carvone.

**Figure S78.** Emission spectra of Eu-FDA-R with 60% ee S-carvone.

**Figure S79.** Emission spectra of Eu-FDA-S with -60% ee S-carvone.

**Figure S80.** Emission spectra of Eu-FDA-S with 0% ee S-carvone.

**Figure S81.** Emission spectra of Eu-FDA-S with 60% ee S-carvone.

**Figure S82.** Emission spectra of Eu-FDA-R with -60% ee quinidine.

**Figure S83.** Emission spectra of Eu-FDA-R with 0% ee quinidine.

**Figure S84.** Emission spectra of Eu-FDA-R with 60% ee quinidine.

**Figure S85.** Emission spectra of Eu-FDA-S with -60% ee quinidine.

**Figure S86.** Emission spectra of Eu-FDA-S with 0% ee quinidine.

**Figure S87.** Emission spectra of Eu-FDA-S with 60% ee quinidine.

**Sensing Mechanism**

**Figure S88.** PXRD patterns before and after sensing.

**Figure S89.** IR spectra before and after sensing.

**Figure S90.** Emission spectra of Eu-FDA-R/S and UV-vis spectra of the analytes.

**Figure S91.** UV-vis spectra of Eu-FDA-R/S and the analytes.

**Figure S92.** HOMO and LUMO energy levels of the linkers and the analytes.

**Figure S93.** ^1^H NMR spectra of Eu-FDA-R/S with 2-amino-1-butanol.

**Figure S94.** ^1^H NMR spectra of Eu-FDA-R/S with 2-methylpiperazine.

**Figure S95.** ^1^H NMR spectra of Eu-FDA-R/S with carvone.

**Figure S96.** ^1^H NMR spectra of Eu-FDA-R/S with quinine and quinidine.

**References**

[1]	J. Zhou, H. Li, H. Zhang, H. Li, W. Shi, P. Cheng, A bimetallic lanthanide metal-organic material as a self-calibrating color-gradient luminescent sensor. *Adv. Mater.* **2015**, *27*, 7072-7077.

[2]	Z. Han, K.-Y. Wang, R.-R. Liang, Y. Guo, Y. Yang, M. Wang, Y. Mao, J. Huo, W. Shi, H.-C. Zhou Modular construction of multivariate metal-organic frameworks for luminescent sensing. *J. Am. Chem. Soc*. **2025**, *147*, 3866-3873.
